# Age of Information in Wireless Powered Networks in Low SNR Region for Future 5G

**DOI:** 10.3390/e20120948

**Published:** 2018-12-10

**Authors:** Huimin Hu, Ke Xiong, Yu Zhang, Pingyi Fan, Tong Liu, Shaoli Kang

**Affiliations:** 1 School of Computer and Information Technology, Beijing Jiaotong University, Beijing 100044, China; 2 Beijing Key Laboratory of Traffic Data Analysis and Mining, Beijing Jiaotong University, Beijing 100044, China; 3 State Grid Energy Research Institute Co., Ltd., Beijing 102209, China; 4 Department of Electronic Engineering, Tsinghua University, Beijing 100084, China; 5 Beijing Computing Center, Beike Industry Park, Beijing 100094, China; 6 China Academy of Telecommunications Technology, Beijing 100191, China

**Keywords:** age of information, energy harvest, wireless power, block Rayleigh fading channel

## Abstract

Wireless powered communication technology has a great potential to power low-power wireless sensor networks and Internet of Things (IoT) for real-time applications in future 5G networks, where age of information (AoI) plays a very important performance metric. This paper studies the system average AoI of a wireless powered network, where a wireless-powered user harvests energy from a wireless power source (WPS) and then transmits data packets to its access point (AP) by using the harvested energy. The user generates data packets with some probability and adopts the first-come-first-served (FCFS) service policy. For such a system, by using the queuing theory and the probability models, we derive a closed-form expression of the system average AoI. We also formulate an optimization problem to minimize the AoI by optimizing the data packet generating probability, and find its solution by simple calculation and search. Simulation results demonstrate the correctness of our obtained analytical results. It also shows that, when the total distance of the two hops is fixed, the system average AoI increases linearly with the increment of the distance of the first hop, and a smaller data packet generating probability should be selected to match a bigger first-hop distance for achieving a smaller system average AoI. Moreover, a smaller data packet size also contributes to a smaller system average AoI.

## 1. Introduction

Recently, the widespread use of mobile devices and applications has made the real-time information updating applications such as news, weather forecasting and traffic alert more and more popular [1,2,3]. Timely information updating is also becoming more and more critical for real-time monitoring and control systems, including wireless sensor networks (WSNs) and internet of Things (IoT) for temperature and humidity detection in warehouses [4], safety and state monitoring in industrial production lines, embedded equipments in medical care [5], and road condition detection in automatic droving for future 5G systems [6,7]. The common key point of above-mentioned real-time applications is how to guarantee the freshness of the collected data.

Traditionally, delay and throughput are two important and widely adopted indices to evaluate the system performance of communication networks [8,9]. However, they are insufficient to describe the freshness of the data [10]. Therefore, a new metric, referred to as age of information (AoI), has emerged recently, which is defined to characterize the elapsed time since the last received data was generated [11]. The AoI actually describes the freshness of status updates based on time-varying wireless channel transmissions because it can reflect real world constraints condition that the delivery of a status message requires a nonzero and typically random time in the system [12].

In the past several years, AoI has been analyzed and studied in various queuing systems. For instance, the AoI was analyzed for single-source single-server queues in [11], and for M/M/1 first-come-first-served (FCFS) systems with multiple sources in [13]. In [14], AoI was explored for a multi-class M/G/1 queueing system. In [15], a packet deadline was regarded as a control mechanism to study its impact on the average AoI for M/M/1 queueing systems, and in [16], it was found that the packet waiting in queue was replaced if a new packet arrives service discipline is optimal.

Meanwhile, in order to realize the fresh-data transmission and explore the system performance limit, AoI was minimized for single-hop networks, see e.g., [11,14,17,18], and multi-hop networks, see e.g., [19,20], with different system setups. In [19], it was shown that for general system settings (including arbitrary network topology, packet generation times, packet arrival times, and queue buffer sizes), age-optimality can be achieved. In [20], an energy harvesting (EH) assisted two-hop system was studied, where an EH-enabled source collected measurements from a physical phenomenon and then sent updates to a destination with the help of an EH-enabled relay.

As for EH, it is able to power the device by harvesting energy from external environment [21] and has a great potential to be applied to energy-constrained networks including IoT and WSNs in future 5G [22,23,24,25,26,27,28]. EH technologies can be classified into two categories: the natural energy source-based EH and the radio frequency (RF) signal-based EH [29,30,31,32,33,34]. Compared with natural source, RF signal is easier to control and has less environmental limitations to deploy. Therefore, it is becoming more and more popular. Since RF EH-enabled IoT and sensor networks are expected to be widely employed for industrial control, unmanned driving systems and real-time applications, analyzing AoI performance and designing AoI minimized systems for RF-EH wireless networks have attracted increasing interest, see e.g., [35,36]. In [35], the average AoI of the two-way networks, where the slave node is powered by RF EH supply and the uplink average AoI was analyzed. In [36], the authors studied how to design optimal online status update policies to minimize the long-term average AoI, subject to the energy causality constraint at the sensor.

In this paper, we study the average AoI in a wireless powered network, where a wireless-powered user harvests energy from a wireless power source (WPS) and transmits data packets to its access point (AP). The user generates a data packet with some probability *p* in each time block, and the generated data are stored in an infinite buffer queue. FCFS service policy is employed, using the queuing theory and the probability model. We derive a closed-form expression of the average AoI for such a system. Numerical results are provided to discuss the system AoI performance. It is observed that there is an optimal *p* such that the AoI reaches minimal. Therefore, we formulate an optimal problem to minimize the AoI to find the optimal *p*. Since the problem is non-convex, we use the one-dimensional search to find the optimal *p*. Moreover, with other parameters being fixed, we find that the minimal average AoI linearly increase with the increment of packet size. In addition, we also analyze the effects of other factors on the average AoI. It is found that, when the total distance of the two hops is fixed, the system average AoI increases linearly with the increment of the distance of the first hop, and a smaller data packet generating probability should be selected to match a bigger first-hop distance for achieving a smaller system average AoI. Moreover, a smaller data packet size also contributes to a smaller system average AoI.

The rest of the paper is organized as follows. In Section 2, we present the system model, including the channel model, the data transmission model and the energy transfer model. In Section 3, we derive an explicit expression of the average AoI. Section 4 discusses the system AoI performance via simulations. Section 5 summarizes the paper with some conclusions.

## 2. System Model

**Figure 1:**
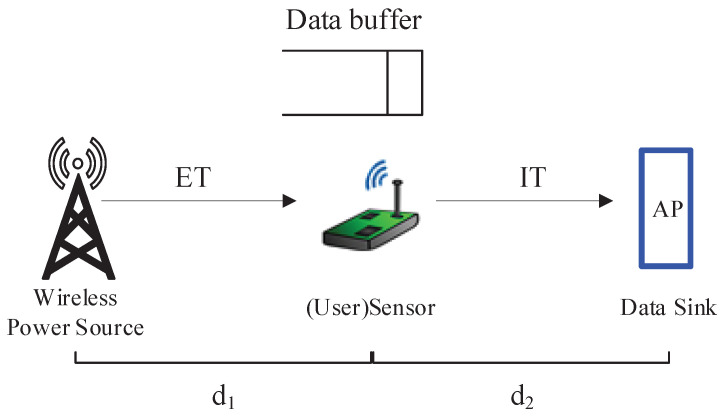
An illustration of the wireless powered communication system.

We consider a wireless powered network, as depicted in Figure 1, where a user (e.g., a sensor node) desires to transmit its data to its AP (e.g., a sink node). Since the user is lack of energy, it has to harvest energy from a WPS that deployed in the system and used to charge the wireless devices via wireless power transfer. Our considered network model can be regarded as a basic component of complex networks. For example, when multiple nodes are deployed, by using time division multiple access(TDMA) or frequency division multiple access (FDMA), the complex network can be decomposed into multiple point-to-point networks [37,38,39], each of which is like our considered model.

It is assumed that the system works in a discrete time manner. That is, the time is divided into blocks with equal interval $T_{b}$. The time period from the epoch *n* to the epoch n+1 is referred to as the time block *n*. Block Rayleigh fading channel model from WPS to the user and from the user to its AP is assumed, so that the channel coefficient can be considered as a constant in each time block, and it may vary from one time block to the next for information transmission. Let $h_{1}\left[n\right]$ and $h_{2}\left[n\right]$ be the channel coefficients of the links from the WPS to the user and from the user to the AP, respectively, associated with time block *n*. The corresponding power gains $|h_{1}{|}^{2}$ and $|h_{2}{|}^{2}$ follow the exponential distribution, which can be expressed by

(1) $$f_{|h_{1}{|}^{2}}\left(x\right)=\lambda_{1}e^{-\lambda_{1}x},$$

and

(2) $$f_{|h_{2}{|}^{2}}\left(x\right)=\lambda_{2}e^{-\lambda_{2}x},$$

where $\lambda_{1}$ and $\lambda_{2}$ are the exponential distribution parameters.

In each block, the user generates a data packet with size of δ bits randomly with a certain probability *p*, and the generated data packets are first stored in a data buffer, and then transmitted with the FCFS policy. Denote the distance between the WPS and the user and between the user and the AP to be $d_{1}$ and $d_{2}$, respectively. Let the transmit power of the WPS be $P_{w}$. If in time block *n*, the user performs energy harvesting, the energy received at the user from the WPS in time block *n* is given by

(3) $$E\left[n\right]=\mu |h_{1}{\left[n\right]|}^{2}P_{w}T_{b},$$

where η is the energy transfer efficiency and $\mu =\frac{\eta }{d_{1}^{\alpha }}$. α is the pass loss factor.

Since the energy transfer efficiency η is less than one and the received power is also relatively small due to pass loss, the user may take several blocks to harvest and accumulate energy to complete a block of transmission. The energy accumulated at the user within *j* blocks is

(4) $$e_{j}=\sum_{i=1}^{j}E\left[i\right]=\mu P_{w}T_{b}\sum_{i=1}^{j}|h_{1}{\left[i\right]|}^{2}.$$

According to Equation (1), $|h_{1}{|}^{2}$ follows exponential distribution with parameter $\lambda_{1}$, so $e_{j}$ follows Erlang $\left(j,\frac{\lambda_{1}}{\mu P_{w}T_{b}}\right)$ distribution, i.e.,

(5) $$f_{e_{j}}=\frac{v^{j}x^{j-1}e^{-vx}}{(j-1)!},$$

where $v=\frac{\lambda_{1}}{\mu P_{w}T_{b}}$.

It is assumed that the energy transmission and data transmission are over orthogonal frequency bands. Let the transmit power of the user be $P_{u}$. Assume that the received signals are suffered from additive white Gaussian noise. If in time block *n*, the user performs data transmission, the data size can be delivered in block *n* is (see Endnote [40]—which refers to Reference [35,41])

$$c\left[n\right]=T_{b}B\log\left(1+\frac{|h_{2}{\left[n\right]|}^{2}P_{u}}{BN_{0}}\right),$$

where *B* is the system bandwidth and $N_{0}=n_{0}d_{2}^{\alpha }$ with $n_{0}$ denoting the noise spectral density.

For such a system, our goal is to analyze its average AoI performance in fading channels.

## 3. AOI Analysis

In IoT and sensor networks, the transmit power of the devices is usually very low, so that the received energy from the transmitted signals of WPS at the user is relatively very small. Therefore, we analyze the system AoI performance in the low SNR regime, where c[n] can be approximated by

(6) $$c\left[n\right]\approx \frac{|h_{2}{\left[n\right]|}^{2}P_{u}T_{b}}{N_{0}}.$$

### 3.1. Preliminary Analysis

Denote the number of time blocks required to complete the transmission of one data packet with size of δ to be $N_{I}$. Define the probability of successfully completing a packet transmission with *j* time blocks to be $p_{j}^{n_{i}}$. When the user accumulates sufficient energy, we obtain the following theoretical results.

**Proposition** **1.** *The probability of successfully completing a packet transmission with j time blocks is*

(7) $$\begin{array}{c} p_{j}^{n_{i}}=\frac{\theta^{j-1}}{(j-1)!}e^{-\theta }, \end{array}$$

*where j=1,2,3,⋯,n and $\theta =\frac{\lambda_{2}N_{0}\delta }{P_{u}T_{b}}$.*

**Proof.**  According to the definition of $p_{j}^{n_{i}}$ and [42], it can be expressed that

$$\begin{array}{cc} p_{j}^{n_{i}} & =\mathrm{Pr}\left\{N_{I}=j\right\}\\ & =\mathrm{Pr}\left\{\sum_{i=1}^{j-1}c\left[i\right]<\delta ,\sum_{i=1}^{j}c\left[i\right]\geq \delta \right\}\\ & =\int_{0}^{\delta }f_{C}\left(x\right)dx\int_{\delta -x}^{\infty }f_{c}\left(x\right)dx, \end{array}$$

where $f_{C}\left(x\right)$ and $f_{c}\left(x\right)$ are probability density function (pdf) of $\sum_{i=1}^{j-1}c\left[i\right]$ and c[i], respectively.

Since $|h_{2}{|}^{2}$ follows exponential distribution, from Equation (6), c[n] obeys exponential distribution. Thus, the cumulative distribution function (CDF) of c[n] is

$$\begin{array}{cc} F_{c}\left(x\right) & =\mathrm{Pr}\left(c<x\right)=\mathrm{Pr}\left(|h_{2}{|}^{2}<\frac{N_{0}}{P_{u}T_{b}}x\right)\\ & =\int_{0}^{\frac{N_{0}x}{P_{u}T_{b}}}\lambda_{2}e^{-\lambda_{2}y}dy=1-e^{-(\frac{\lambda_{2}N_{0}}{P_{u}T_{b}})x}. \end{array}$$

Furthermore, it can be inferred that $\sum_{i=1}^{j}c\left[i\right]$ follows the Gamma distribution. Thus, the pdf of $\sum_{i=1}^{j}c\left[i\right]$ is given by

$$f_{C}\left(x\right)=\frac{{\left(\frac{\lambda_{2}N_{0}}{P_{u}T_{b}}\right)}^{\left(j-1\right)}}{\Gamma \left(j-1\right)}x^{\left(j-2\right)}e^{-(\frac{\lambda_{2}N_{0}}{P_{u}T_{b}})x},$$

where $\Gamma (j-1)=\int_{0}^{\infty }e^{-t}t^{j-2}dt$ is the Gamma function. Thus,

$$\begin{array}{cc} p_{j}^{n_{i}} & =\int_{0}^{\delta }f_{C}\left(x\right)dx\int_{\delta -x}^{\infty }f_{c}\left(x\right)dx\\ & =\int_{0}^{\delta }\frac{{\left(\frac{\lambda_{2}N_{0}}{P_{u}T_{b}}\right)}^{\left(j-1\right)}}{\Gamma \left(j-1\right)}x^{\left(j-2\right)}e^{-(\frac{\lambda_{2}N_{0}}{P_{u}T_{b}})x}dx\int_{\delta -x}^{\infty }f_{c}\left(x\right)dx\\ & =\frac{({\frac{\lambda_{2}N_{0}\delta }{P_{u}T_{b}})}^{j-1}}{(j-1)!}e^{-\frac{\lambda_{2}N_{0}\delta }{P_{u}T_{b}}}, \end{array}$$

with $\theta =\frac{\lambda_{2}N_{0}\delta }{P_{u}T_{b}}$. Therefore, we arrive at Proposition 1. □

**Proposition** **2.** *The probability generating function (PGF) and the expectation of $N_{I}$ are*

(8) $$G_{N_{I}}\left(z\right)=ze^{\theta (z-1)},$$

*and*

(9) $$E(N_{I})=1+\theta ,$$

*respectively.*

**Proof.**  In terms of the definition of PGF, we have

$$\begin{array}{cc} G_{N_{I}}\left(z\right) & =E\left(z^{n_{i}}\right)=\sum_{j=0}^{\infty }z^{j}\mathrm{Pr}\left\{N_{I}=j\right\}\\ & =\sum_{j=0}^{\infty }z^{j}\frac{\theta^{j-1}}{(j-1)!}e^{-\theta }\\ & =ze^{\theta (z-1)}. \end{array}$$

By the property of PGF, we have

$$\begin{array}{c} E\left(N_{I}\right)=\lim_{z\to 1^{-}}G_{N_{I}}^{{}^{\prime }}\left(z\right)=1+\theta . \end{array}$$

 □

In our model, the harvested energy is allowed to be used for information transmission in the same block. As long as the accumulated energy at the user is greater than or equal to $P_{u}T_{b}$, it performed a block of transmission. Suppose the number of blocks that need to harvest energy for the user to complete a block of transmission is $N_{E}$. Since the energy transfer efficiency η is less than one and the received power is also relatively small due to pass loss, the user may take several blocks to harvest energy to complete a block of transmission, i.e., $N_{E}\geq 1$. As a result, the transmitter start information delivering at time block *j*, if

$$\left\{e_{j-1}<P_{u}T_{b},e_{j}\geq P_{u}T_{b}\right\},\;\mathrm{for}\;\;j=1,2,3,\cdots .$$

Therefore, the actual time required to complete a block of transmission is $N_{E}=j.$ Without loss of generality, we suppose $P_{w}=\beta P_{u}$, with β>0. In terms of Equations (4) and (5), the probability $\mathrm{Pr}\{N_{E}=j\}$ of $N_{E}=j$ can be given by

(10) $$\begin{array}{cc} \mathrm{Pr}\{N_{E}=j\} & =\mathrm{Pr}\{e_{j-1}<P_{u}T_{b},e_{j}\geq P_{u}T_{b}\}\\ & =\int_{0}^{P_{u}T_{b}}f_{e_{j}}\left(x\right)dx\int_{P_{u}T_{b}-x}^{\infty }f_{e_{1}}\left(y\right)dy\\ & =\frac{{\left(\frac{\lambda_{1}}{\beta \mu }\right)}^{j-1}}{(j-1)!}e^{-(\frac{\lambda_{1}}{\beta \mu })},\;\;\mathrm{for}\;\;j=1,2,3,\cdots . \end{array}$$

Let the service time of the *k*th data packet is $T_{k}^{\left(S\right)}$, which represents the time for harvesting energy and the time for transmitting data.

**Lemma** **1.** *The probability that j time blocks are needed to actually transfer a data packet is*

(11) $$\begin{array}{cc} p_{j}^{s} & =\mathrm{Pr}\{T_{k}^{\left(S\right)}=j\}\\ & =\frac{{\left(\frac{\left(1+\theta \right)\lambda_{1}}{\beta \mu }\right)}^{j-1}}{(j-1)!}e^{-\frac{\left(1+\theta \right)\lambda_{1}}{\beta \mu }}. \end{array}$$

**Proof.**  When the user accumulates sufficient energy, in terms of Equation (9), the average time to transmit a data packet is 1+θ. Since the user needs $N_{E}$ time blocks to harvest energy to perform a block of transmission, that is, $T_{k}^{\left(S\right)}$ contains 1+θ independent and identically distributed variables $N_{E}\left(i\right)$, according to Equation (10), one can derive Equation (11). □

**Proposition** **3.** *The probability generating function (PGF) of $T_{k}^{\left(S\right)}$ is*

(12) $$G_{T_{k}^{\left(S\right)}}\left(z\right)=ze^{\left(z-1\right)\frac{\left(1+\theta \right)\lambda_{1}}{\beta \mu }},$$

*and the expectation of $T_{k}^{\left(S\right)}$ is*

(13) $$E\left(T_{k}^{\left(S\right)}\right)=1+\frac{\left(1+\theta \right)\lambda_{1}}{\beta \mu }.$$

**Proof.**  In terms of the definition of PGF, we have

$$\begin{array}{cc} G_{T_{k}^{\left(S\right)}}\left(z\right) & =E\left(z^{T}\right)=\sum_{j=0}^{\infty }z^{j}\mathrm{Pr}\left\{T_{k}^{S}=j\right\}\\ & =\sum_{j=0}^{\infty }z^{j}\frac{{\left(\frac{\left(1+\theta \right)\lambda_{1}}{\beta \mu }\right)}^{j-1}}{(j-1)!}e^{-\frac{\left(1+\theta \right)\lambda_{1}}{\beta \mu }}\\ & =ze^{\left(z-1\right)\frac{\left(1+\theta \right)\lambda_{1}}{\beta \mu }}. \end{array}$$

By the property of PGF, we have

$$\begin{array}{c} E\left(T_{k}^{\left(S\right)}\right)=\lim_{z\to 1^{-}}G_{T_{k}^{\left(S\right)}}^{{}^{\prime }}\left(z\right)=1+\frac{\left(1+\theta \right)\lambda_{1}}{\beta \mu }. \end{array}$$

 □

### 3.2. Description of System Average AoI

The AoI is defined as the time elapsed since the last received packet was generated [11], which is used to measure the freshness of information at the destination. We assume that the most recently received data packet at AP in block *n* was generated at the time U[n]. Therefore, the AoI in block *n*, can be expressed by

(14) Δ[n]=n−U[n].

**Figure 2:**
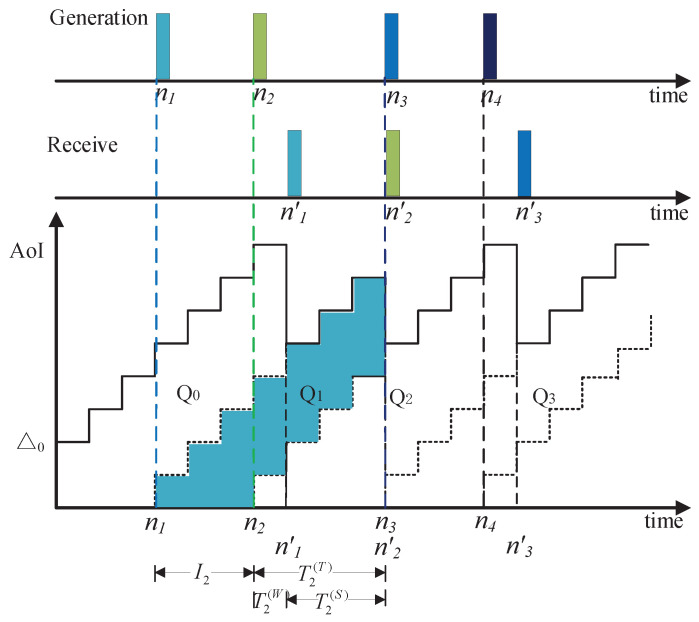
An illustration of a sample evolution of AoI versus time blocks.

Figure 2 illustrates a sample evolution of AoI versus time blocks with initial age $\Delta_{0}$, i.e., $\Delta \left[0\right]=\Delta_{0}$. Since time is discrete, the AoI is constant within each block and varies from a block to next block. Let $n_{k}$ denote the generation time of the *k*th data packet, and $n_{k}^{\prime }$ denote the time when the *k*th data packet is completely transmitted. The number of blocks from the generation of a data packet to the completion of the transmission is $T_{k}^{\left(T\right)}$, i.e.,

$$T_{k}^{\left(T\right)}=n_{k}^{\prime }-n_{k},$$

which is called the system time. The interval time between the generation of data packet k−1 and data packet *k* is set as $I_{k}$. That is,

$$I_{k}=n_{k}-n_{k-1}.$$

Let $T_{k}^{\left(W\right)}$ denote the waiting time of data packet *k*. The time of a data packet in system is also equal to the sum of waiting time $T_{k}^{\left(W\right)}$ and service time $T_{k}^{\left(S\right)}$, i.e.,

$$\;\;\;\;\;\;\;\;\;\;\;\;\;\;\;\;\;\;T_{k}^{\left(T\right)}=T_{k}^{\left(W\right)}+T_{k}^{\left(S\right)}.\;\;\;\;\;\;\;\;\;\;\;\;\;\;\;\;\left(*\right)$$

It is observed that the AoI increases linearly in time and is reset to a smaller value when a data packet is received. That is, at $n_{k}^{\prime }$, the AoI is reset to $\Delta \left[n\right]=n_{k}^{\prime }-n_{k}$. Over a period of *N* blocks where *K* data packets are delivered, the average AoI is defined as

(15) $$\bar{\Delta }=\frac{1}{N}\sum_{n=1}^{N}\Delta \left[n\right].$$

As illustrated in Figure 2, the average AoI of system can be calculated as the average area of the blue graphic $Q_{k}$, i.e.,

(16) $$\bar{\Delta }=\lim_{N\to \infty }\frac{1}{N}\left(Q_{0}+\sum_{k=1}^{K-1}Q_{k}+R_{K}\right),$$

where $R_{K}=\frac{1}{2}T_{K}^{\left(T\right)}\left(T_{K}^{\left(T\right)}+1\right)$.

In the following, we shall discuss how to derive a closed-form exprtession of the average AoI $\bar{\Delta }$.

### 3.3. Closed-Form Expression of $\bar{\Delta }$

From Figure 2, one can see that the area of $Q_{0}$ and $R_{K}$ are limited, with large enough *N*, $\frac{Q_{0}}{N}$ and $\frac{R_{k}}{N}$ are close to zero. Therefore, $\bar{\Delta }$ can be approximatively give by

(17) $$\begin{array}{c} \bar{\Delta }=\lim_{N\to \infty }\frac{1}{N}\sum_{k=1}^{K-1}Q_{k}=\lim_{N\to \infty }\frac{K-1}{N}\frac{1}{K-1}\sum_{k=1}^{K-1}Q_{k}=pE\left(Q_{k}\right). \end{array}$$

**Figure 3:**
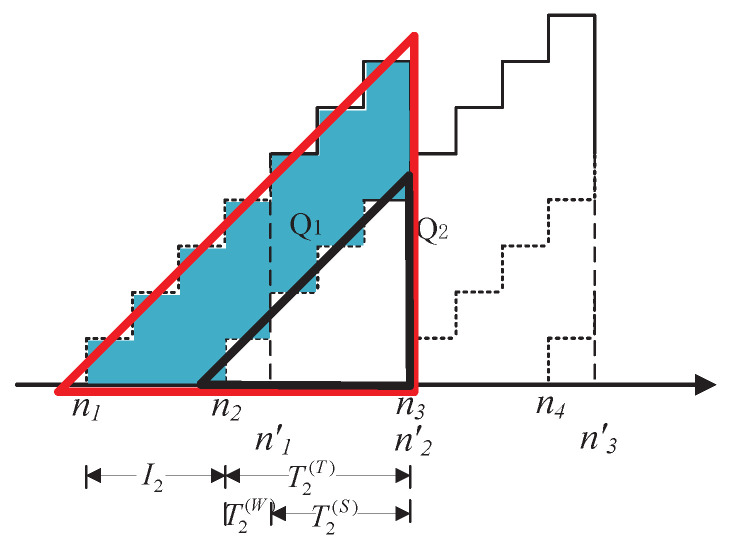
An illustration of an example of calculating AoI.

Moreover, one can see that the area of $Q_{k}$ is the difference of the area of the large red triangle minus the area of the small black triangle, as illustrated in Figure 3. That is,

(18) $$\begin{array}{cc} E(Q_{k}) & =E\left(\frac{1}{2}{\left(T_{k}^{\left(T\right)}+I_{k}+\frac{1}{2}\right)}^{2}-\frac{1}{2}{\left(T_{K}^{\left(T\right)}+\frac{1}{2}\right)}^{2}\right)\\ & =E\left(\frac{1}{2}I_{k}\left(I_{k}+1\right)+I_{k}T_{K}^{\left(T\right)}\right)\\ & =\frac{1}{2}E\left(I_{k}\right)+\frac{1}{2}E\left(I_{k}^{2}\right)+E\left(I_{k}T_{K}^{\left(T\right)}\right). \end{array}$$

Now, we begin to drive the explicit expressions of $E\left(I_{k}\right)$, $E\left(I_{k}^{2}\right)$ and $E\left(I_{k}T_{K}^{\left(T\right)}\right)$ as follows.

#### 3.3.1. Expressions of $E\left(I_{k}\right)$ and $E\left(I_{k}^{2}\right)$

Since *p* is the generation rate of the data packet at the user, i.e., data generating with probability *p* in each block. Therefore, the inter arrival time $I_{k}$ follows geometric distribution. $I_{k}=j$ means that in the *j*-th block data packet is successfully generated, but was not generated in the previous j−1 consecutive blocks. Thus,

$$\mathrm{Pr}\left\{I_{k}=j\right\}={\left(1-p\right)}^{j-1}p,\;\;j=1,2,\cdots .$$

As a result,

(19) $$E\left(I_{k}\right)=\frac{1}{p},$$

and

(20) $$E\left(I_{k}^{2}\right)=\frac{2-p}{p^{2}}.$$

#### 3.3.2. Expressions of $E\left(I_{k}T_{K}^{\left(T\right)}\right)$

Let $Y_{k}$ be the number of data packets generated during the system time of the *k*th data packet. It is also a random variable. To describe it, we define $\{Y_{k}\}$ as an independent and identically distributed random sequence. The probability of generating *j* data packets during the service time of a data packet can be given by

$$p_{j}^{y}=\mathrm{Pr}\left\{Y_{k}=j\right\},$$

where j=0,1,2,⋯ and $\sum_{j=0}^{\infty }p_{j}^{y}=1$. Let $L_{k}$ be the number of data packets in the queue before servicing the *k*th data packet. Thus, we have

(21) $$L_{k+1}=\left\{\begin{array}{c} L_{k}-1+Y_{k},\;L_{k}\geq 1,\\ Y_{k},\;\;\;L_{k}=0. \end{array}\right.$$

**Figure 4:**
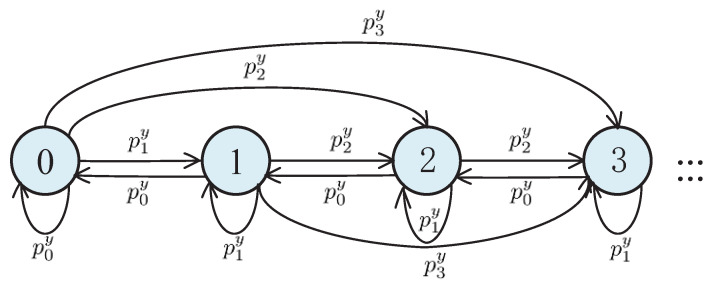
State transition diagram of Markov chain $\{L_{k},k\geq 0\}.$

By regarding $\left\{L_{k}\right\}$ as a state of the system, it can be expressed by a Markov chain. Its state transition diagram is shown in Figure 4, where the numbers in the figure represents the different status (i.e., value of $L_{k}$), and the probability on the curve represents the transition probability from one state to the next. The transition probability is obtained as follows:

$$\begin{array}{cc} p_{00}^{y} & =\mathrm{Pr}\left(L_{k+1}=0|L_{k}=0\right)=\mathrm{Pr}\left(Y_{k}=0\right)=p_{0}^{y},\\ p_{01}^{y} & =\mathrm{Pr}\left(L_{k+1}=1|L_{k}=0\right)=\mathrm{Pr}\left(Y_{k}=1\right)=p_{1}^{y},\\ p_{02}^{y} & =\mathrm{Pr}\left(L_{k+1}=2|L_{k}=0\right)=\mathrm{Pr}\left(Y_{k}=2\right)=p_{2}^{y},\\ p_{03}^{y} & =\mathrm{Pr}\left(L_{k+1}=3|L_{k}=0\right)=\mathrm{Pr}\left(Y_{k}=3\right)=p_{3}^{y},\\ p_{10}^{y} & =\mathrm{Pr}\left(L_{k+1}=0|L_{k}=1\right)=\mathrm{Pr}\left(L_{k}-1+Y_{k}=0|L_{k}=1\right)=\mathrm{Pr}\left(Y_{k}=0\right)=p_{0}^{y},\\ p_{11}^{y} & =\mathrm{Pr}\left(L_{k+1}=1|L_{k}=1\right)=\mathrm{Pr}\left(L_{k}-1+Y_{k}=1|L_{k}=1\right)=\mathrm{Pr}\left(Y_{k}=1\right)=p_{1}^{y},\\ p_{12}^{y} & =\mathrm{Pr}\left(L_{k+1}=2|L_{k}=1\right)=\mathrm{Pr}\left(L_{k}-1+Y_{k}=2|L_{k}=1\right)=\mathrm{Pr}\left(Y_{k}=2\right)=p_{2}^{y},\\ p_{13}^{y} & =\mathrm{Pr}\left(L_{k+1}=3|L_{k}=1\right)=\mathrm{Pr}\left(L_{k}-1+Y_{k}=3|L_{k}=1\right)=\mathrm{Pr}\left(Y_{k}=3\right)=p_{3}^{y}.\\ & ... \end{array}$$

By doing so, the transition probability associated with every link in Figure 4 is determined.

**Proposition** **4.** *The probability distribution and the PGF of Y are respectively given by,*

(22) $$\begin{array}{c} p_{j}^{y}=\left(1-p\right)e^{-\frac{\left(1+\theta \right)\lambda_{1}}{\beta \mu }p}\frac{{\left(\frac{\left(1+\theta \right)\lambda_{1}}{\beta \mu }p\right)}^{j}}{j!}+pe^{-\frac{\left(1+\theta \right)\lambda_{1}}{\beta \mu }p}\frac{{\left(\frac{\left(1+\theta \right)\lambda_{1}}{\beta \mu }p\right)}^{j-1}}{(j-1)!}, \end{array}$$

*and*

(23) $$G_{Y}\left(z\right)=\left(1-p+pz\right)e^{\frac{\left(1+\theta \right)\lambda_{1}}{\beta \mu }p\left(z-1\right)}.$$

**Proof.**  See Appendix A. □

In terms of Foster’s Theorem of [43], when the data arrival rate less than the service rate, the system is stable. That is, $p\left(1+\frac{\left(1+\theta \right)\lambda_{1}}{\beta \mu }\right)<1$. Therefore, we discuss the case when $p\left(1+\frac{\left(1+\theta \right)\lambda_{1}}{\beta \mu }\right)<1$, where $\left(1+\frac{\left(1+\theta \right)\lambda_{1}}{\beta \mu }\right)$ is actually the service time of a data packet. In this case, Markov chain $\left\{L_{k}\right\}$ has a stationary distribution $\pi =(\pi_{0},\pi_{1},...)$ with

$$\pi_{j}=\lim_{k\to \infty }\mathrm{Pr}\left\{L_{k}=j\right\}.$$

Thus, we can obtain the following Proposition 5.

**Proposition** **5.** *When $p\left(1+\frac{\left(1+\theta \right)\lambda_{1}}{\beta \mu }\right)<1$, the PGF of $L_{k}$ is given by*

(24) $$G_{L_{k}}\left(z\right)=\frac{\left(1-p-\frac{\left(1+\theta \right)\lambda_{1}}{\beta \mu }p\right)\left(1-p+pz\right)\left(1-z\right)}{1-p+pz-ze^{\frac{\left(1+\theta \right)\lambda_{1}}{\beta \mu }p\left(1-z\right)}}.$$

**Proof.**  See Appendix B. □

**Proposition** **6.** *The PGF of the system time of each data packet is given by*

(25) $$G_{T}\left(z\right)=\frac{\left(1-p-\frac{\left(1+\theta \right)\lambda_{1}}{\beta \mu }p\right)z\left(1-z\right)}{pz-\left(z-1+p\right)e^{\frac{\left(1+\theta \right)\lambda_{1}}{\beta \mu }\left(1-z\right)}}.$$

**Proof.**  See Appendix C. □

**Lemma** **2.** *The average system time is*

(26) $$E\left(T_{k}^{\left(T\right)}\right)=1+\frac{\left(1+\theta \right)\lambda_{1}}{\beta \mu }+\frac{2p\frac{\left(1+\theta \right)\lambda_{1}}{\beta \mu }+{\left(\frac{\left(1+\theta \right)\lambda_{1}}{\beta \mu }\right)}^{2}p}{2(1-p-\frac{\left(1+\theta \right)\lambda_{1}}{\beta \mu }p)}.$$

**Proof.**  By the property of PGF, we can obtain

$$\begin{array}{cc} E(T_{k}^{\left(T\right)}) & =\lim_{z\to 1^{-}}G_{T}^{{}^{\prime }}\left(z\right)\\ & =\frac{{\left(\frac{\left(1+\theta \right)\lambda_{1}}{\beta \mu }\right)}^{2}p+2\frac{\left(1+\theta \right)\lambda_{1}}{\beta \mu }p+2p-2\frac{\left(1+\theta \right)\lambda_{1}}{\beta \mu }-2}{2(\frac{\left(1+\theta \right)\lambda_{1}}{\beta \mu }p+p-1)}\\ & =1+\frac{\left(1+\theta \right)\lambda_{1}}{\beta \mu }+\frac{2p\frac{\left(1+\theta \right)\lambda_{1}}{\beta \mu }+{\left(\frac{\left(1+\theta \right)\lambda_{1}}{\beta \mu }\right)}^{2}p}{2(1-p-\frac{\left(1+\theta \right)\lambda_{1}}{\beta \mu }p)}. \end{array}$$

 □

Recall Equation (∗), i.e., the system time of data packet *k*. Note that, when the *k*th data packet is generated at the user, if the (k−1)th data packet has completed the service, $T_{k}^{\left(W\right)}=0$. In this case, the waiting time for the data packet is $T_{k}^{\left(W\right)}=\max\left(0,T_{k-1}^{\left(T\right)}-I_{k}\right)$. As $T_{k}^{\left(W\right)}$ and $I_{k}$ are not independent of each other, and $T_{k}^{\left(S\right)}$ is independent of $I_{k}$, one has

(27) $$\begin{array}{cc} E\left(I_{k}T_{k}^{\left(T\right)}\right) & =E\left(I_{k}\left(T_{k}^{\left(W\right)}+T_{k}^{\left(S\right)}\right)\right)\\ & =E\left(I_{k}\right)E\left(T_{k}^{\left(S\right)}\right)+E\left(I_{k}T_{k}^{\left(W\right)}\right). \end{array}$$

**Proposition** **7.** *For the considered system,*

(28) $$\begin{array}{c} E\left(I_{k}T_{k}^{\left(W\right)}\right)=\frac{\left(-2+4p+3\frac{\left(1+\theta \right)\lambda_{1}}{\beta \mu }p\right)\frac{\left(1+\theta \right)\lambda_{1}}{\beta \mu }}{2p(1-p-\frac{\left(1+\theta \right)\lambda_{1}}{\beta \mu }p)}+\frac{\left(1-p-\frac{\left(1+\theta \right)\lambda_{1}}{\beta \mu }p\right)\left(e^{\frac{\left(1+\theta \right)\lambda_{1}}{\beta \mu }p}-1\right)}{p^{2}}. \end{array}$$

**Proof.**  See Appendix D. □

**Theorem** **1.** *When $p\left(1+\frac{\left(1+\theta \right)\lambda_{1}}{\beta \mu }\right)<1$, the average AoI is given by*

(29) $$\begin{array}{c} \bar{\Delta }=\frac{1}{p}+\left(1+\frac{\left(1+\theta \right)\lambda_{1}}{\beta \mu }\right)+\frac{\left(-2+4p+3\frac{\left(1+\theta \right)\lambda_{1}}{\beta \mu }p\right)\frac{\left(1+\theta \right)\lambda_{1}}{\beta \mu }}{2(1-p-\frac{\left(1+\theta \right)\lambda_{1}}{\beta \mu }p)}+\frac{\left(1-p-\frac{\left(1+\theta \right)\lambda_{1}}{\beta \mu }p\right)\left(e^{\frac{\left(1+\theta \right)\lambda_{1}}{\beta \mu }p}-1\right)}{p}, \end{array}$$

*where $\theta =\frac{\lambda_{2}N_{0}\delta }{P_{u}T_{b}}$.*

**Proof.**  Combining Equations (13), (17)–(20) and (28), one can get the expression for $\bar{\Delta }$. □

Furthermore, from Theorem 1, we can get the following two corollaries.

**Corollary** **1.** *When $p\left(1+\frac{\left(1+\theta \right)\lambda_{1}}{\beta \mu }\right)\to 0$, (i.e., p→0), $\bar{\Delta }\to \infty$.*

**Proof.**  Let p=ϵ, where ϵ>0 is a small enough number. Equation (29) can be approximately written as

(30) $$\bar{\Delta }\approx \lim_{\epsilon \to 0}\frac{1}{\epsilon }+\left(1+\frac{\left(1+\theta \right)\lambda_{1}}{\beta \mu }\right).$$

Since

$$\lim_{\epsilon \to 0}\frac{1}{\epsilon }+\left(1+\frac{\left(1+\theta \right)\lambda_{1}}{\beta \mu }\right)=\infty ,$$

we arrive at Corollary 1. □

**Corollary** **2.** *When $p\left(1+\frac{\left(1+\theta \right)\lambda_{1}}{\beta \mu }\right)\to 1$, (i.e., $p\to \frac{1}{1+\frac{\left(1+\theta \right)\lambda_{1}}{\beta \mu }}$), $\bar{\Delta }\to \infty$.*

**Proof.**  Let $p=\frac{1}{1+\frac{\left(1+\theta \right)\lambda_{1}}{\beta \mu }}$ and $1-p\left(1+\frac{\left(1+\theta \right)\lambda_{1}}{\beta \mu }\right)=\epsilon$, where ϵ>0 is a small enough number. Equation (29) can be approximately written as

(31) $$\bar{\Delta }\approx \lim_{\epsilon \to 0}2\left(1+\frac{\left(1+\theta \right)\lambda_{1}}{\beta \mu }\right)+\frac{\frac{\left(1+\theta \right)\lambda_{1}}{\beta \mu }\left(\frac{\left(1+\theta \right)\lambda_{1}}{\beta \mu }+2\right)}{2\left(\frac{\left(1+\theta \right)\lambda_{1}}{\beta \mu }+1\right)}\cdot \frac{1}{\epsilon }.$$

Since

$$\lim_{\epsilon \to 0}2\left(1+\frac{\left(1+\theta \right)\lambda_{1}}{\beta \mu }\right)+\frac{\frac{\left(1+\theta \right)\lambda_{1}}{\beta \mu }\left(\frac{\left(1+\theta \right)\lambda_{1}}{\beta \mu }+2\right)}{2\left(\frac{\left(1+\theta \right)\lambda_{1}}{\beta \mu }+1\right)}\cdot \frac{1}{\epsilon }=\infty ,$$

we arrive at Corollary 2. □

### 3.4. The Minimal Average AoI of the System

Our objective is to minimize the average AoI. The maximum data rate that can make the data packets queue stable is $p_{\max}=\frac{1}{1+\frac{\left(1+\theta \right)\lambda_{1}}{k\mu }}$. Hence, we can formulate the problem as follows:

**Problem** **1.** (32) $$\begin{array}{cc} \min_{p} & \;\bar{\Delta }\\ s.t. & \;0<p<p_{\max}. \end{array}$$

Since it is difficult to theoretically prove that the problem 1 is a convex or non-convex, we cannot directly obtain the optimal solution. Thus, we use the one-dimensional search to find the optimal *p*, and give simulation results to illustrate that there exists an optimal solution for Problem 1 in the next section (see Endnote [44]).

**Fact** **1.** *The average AoI converges to a constant with the increment of $P_{u}$.*

Although it is difficult to prove Fact 1 and the converged constant is also hard to derive mathematically, we may analyze it from the physical perspective. It is known that with the increment of $P_{u}$, the information rate of the second hop is increased, which may decrease the system AoI. However, due to the Shannon capacity theorem, the information rate of the second hop cannot be increased infinitely, so the system AoI cannot be decreased infinitely, which must converge to some value with the increment of $P_{u}$.

## 4. Numerical Results

In this section, numerical simulations are conducted to discuss the system AoI performance. The simulation parameters are set according to [35]. Specifically, the distances between the WPS and the user and between the user and the AP, are set to unit distance, i.e., $d_{1}=d_{2}=1$ meter. The pass loss factor is α=2. The transmit power of the user is set as $P_{u}=0.01$ W, and β=1, i.e., $P_{w}=P_{u}$. The size of data packet is δ=8 bits. The energy transmission efficiency is η=0.6, the system bandwidth is B=1 MHz, the block length is set as $T_{b}=10^{-3}$ s, and the noise spectral density is $n_{0}=4\times 10^{-7}$. The parameters of the channel power gain $|h_{1}{|}^{2}$ and $|h_{2}{|}^{2}$ are set as $\lambda_{1}=3$ and $\lambda_{2}=3$, respectively.

**Figure 5:**
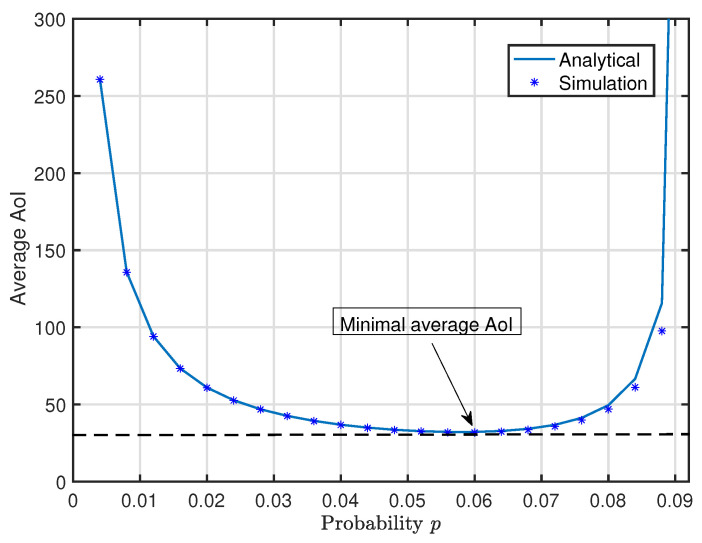
Average age of information versus data generation probability *p*.

As shown in Figure 5, the analytical results match the simulation ones well, which validates the correctness of our analytical results. Moreover, when *p* is close to 0, the average AoI is very large because the interval of data generation $I_{k}$ is very large in this case. When *p* approaches $p_{\max}$, the average AoI also increases to be infinite because, in this case, the waiting time in the queue becomes very long. This observation is consistent with Corollary 1 and 2. It is also observed that there exists a unique optimal *p* such that the system average AoI achieves a minimum.

**Figure 6:**
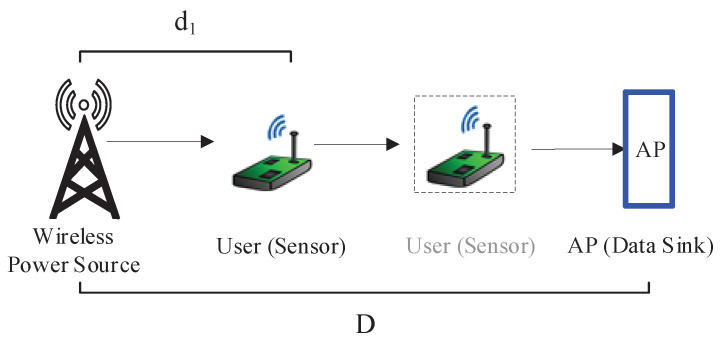
Illustration of the simulation scenario by moving the user from the WPS to the AP with the fixed D.

**Figure 7:**
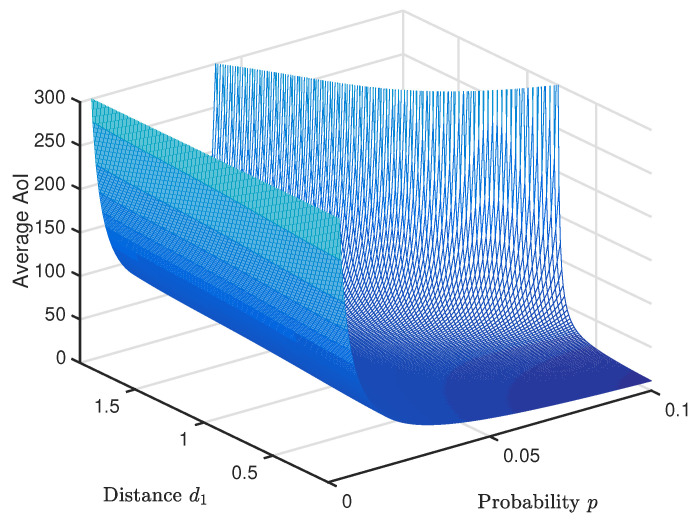
Average age of information versus data generation probability *p* and distance.

To further discuss the system performance, we fix $D=d_{1}+d_{2}$ to be two meters and then increase $d_{1}$ gradually, as illustrated in Figure 6. The simulation results are plotted in a 3D figure as shown in Figure 7. One can observe that with, the increment of $d_{1}$, the system average AoI increases linearly, and the smaller *p* should be selected for the bigger $d_{1}$ in order to achieve the system minimal average AoI.

**Figure 8:**
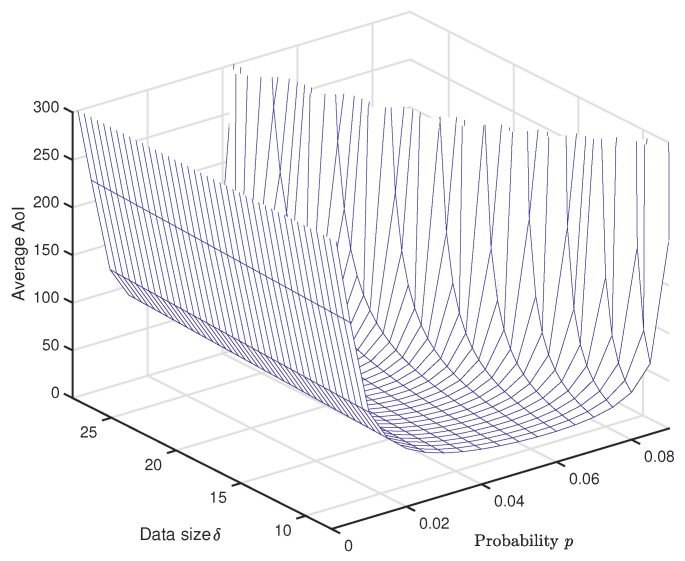
Average age of information versus data generation probability *p* and data size δ.

**Figure 9:**
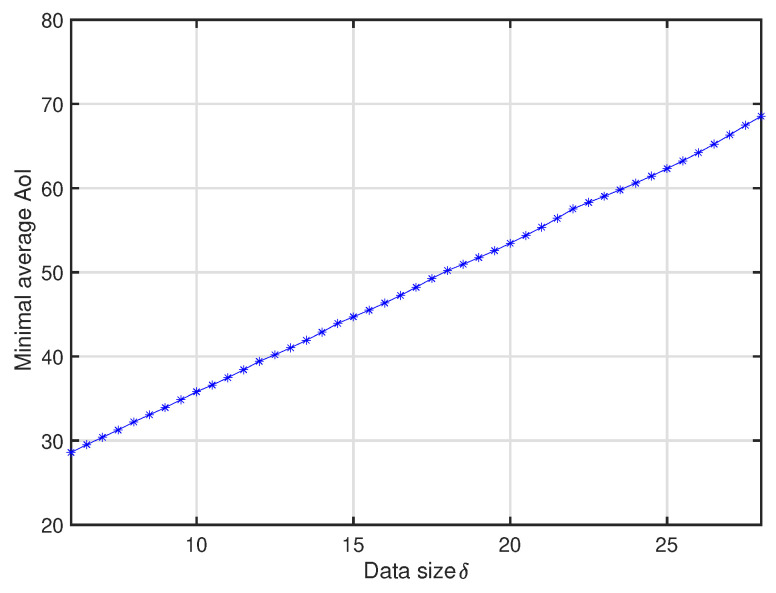
Impact of data size δ on average AoI under optimal *p*.

Figure 8 plots the system average AoI versus data generation probability *p* and data size δ in a 3D figure. One can see that the minimal average AoI increases linearly with the increment of packet size δ, and the smaller *p* should be selected for the larger δ. In order to clearly show this, for each given data size δ, we mark the corresponding minimum AoI and it is plotted versus δ in Figure 9, which can be considered as the lower bound of the system average AoI. This observation also implies that, to keep the data fresh, the smaller packet size is preferred.

**Figure 10:**
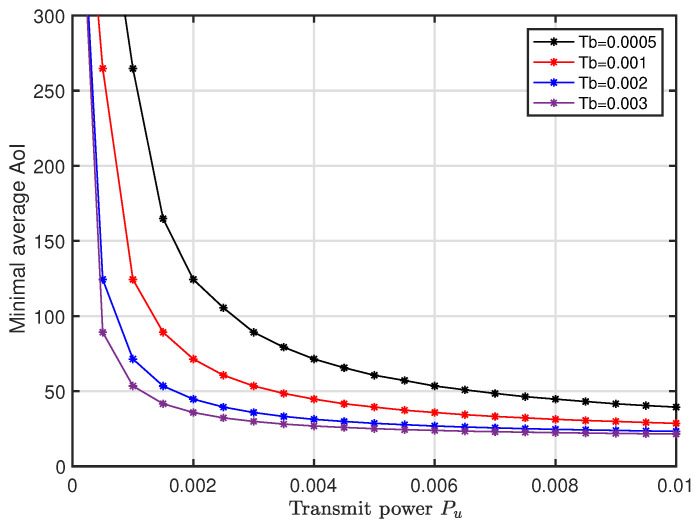
Impact of transmit power $P_{u}$ on average AoI under optimal *p*.

Figure 10 shows the system minimal average AoI versus $P_{u}$ with different configurations of $T_{b}$. It is shown that, for a given block length $T_{b}$, the minimum AoI decreases with increment of $P_{u}$ and then tends to be flat. That is, the average AoI converges to a constant with the increment of $P_{u}$, which is consistent with the result in Fact 1. Moreover, the larger the the block length $T_{b}$ is, the smaller the minimal AoI of the system is.

## 5. Conclusions

In this paper, we studied the system average AoI of a wireless powered communication network, where a wireless powered user harvests energy from a WPS and then transmits data packets to its AP by using the harvested energy. By using the queuing theory and some typical probability models on the channel fading, we derived a closed-form expression of the system average AoI. Some interesting results are obtained, and there exists an optimal generating probability of sensor data (user) *p* such that the AoI reaches a minimum. Simulation results also show that, when the total distance of the two hops is fixed, the system AoI increases linearly with the increment of the distance of the first hop, and a smaller data packet generating probability should be selected to match a bigger first-hop distance for achieving a smaller system average AoI. Moreover, a smaller data packet size of users will result in contributing to a smaller system average AoI.

## Abbreviations

The following abbreviations are used in this manuscript:

AoI	age of information
CDF	cumulative distribution function
EH	energy harvesting
RF	radio frequency
FCFS	first-come-first-served
PDF	probability density function
FDMA	frequency division multiple access
TDMA	time division multiple access
WPS	wireless power source

## Appendix A

**Proof.** According to Equation (11), $\mathrm{Pr}\left\{T_{k}^{\left(S\right)}=m\right\}=p_{m}^{s}$. For the case $Y_{k}=0$, we have

(A1) $$\begin{array}{cc} p_{0}^{y} & =\sum_{m=1}^{\infty }\mathrm{Pr}\left\{T_{k}^{\left(S\right)}=m\right\}\mathrm{Pr}\left\{Y_{k}=0|T_{k}^{\left(S\right)}=m\right\}\\ & =\sum_{m=j}^{\infty }p_{m}^{s}\mathrm{Pr}\left\{Y_{k}=0|T_{k}^{\left(S\right)}=m\right\}\\ & =\sum_{m=j}^{\infty }\frac{{\left(\frac{\left(1+\theta \right)\lambda_{1}}{\beta \mu }\right)}^{m-1}}{(m-1)!}e^{-\frac{\left(1+\theta \right)\lambda_{1}}{\beta \mu }}{\left(1-p\right)}^{m}\\ & =\left(1-p\right)e^{-\frac{\left(1+\theta \right)\lambda_{1}}{\beta \mu }p}, \end{array}$$

For the case $Y_{k}=j\geq 1$, we have

(A2) $$\begin{array}{cc} p_{j}^{y} & =\sum_{m=j}^{\infty }\mathrm{Pr}\left\{T_{k}^{\left(S\right)}=m\right\}\mathrm{Pr}\left\{Y_{k}=j|T_{k}^{\left(S\right)}=m\right\}\\ & =\sum_{m=j}^{\infty }p_{m}^{s}\mathrm{Pr}\left\{Y_{k}=j|T_{k}^{\left(S\right)}=m\right\}\\ & =\sum_{m=j}^{\infty }\frac{{\left(\frac{\left(1+\theta \right)\lambda_{1}}{\beta \mu }\right)}^{m-1}}{(m-1)!}e^{-\frac{\left(1+\theta \right)\lambda_{1}}{\beta \mu }}\frac{m!}{j!(m-j)!}{\left(1-p\right)}^{m-j}p^{j}\\ & =e^{-(\frac{\left(1+\theta \right)\lambda_{1}}{\beta \mu })}\frac{p^{j}}{j!}\sum_{m=0}^{\infty }{\left(\frac{\left(1+\theta \right)\lambda_{1}}{\beta \mu }\right)}^{m+j-1}\frac{m}{m!}{\left(1-p\right)}^{m}\\ & =\left(1-p\right)e^{-\frac{\left(1+\theta \right)\lambda_{1}}{\beta \mu }p}\frac{{\left(\frac{\left(1+\theta \right)\lambda_{1}}{\beta \mu }p\right)}^{j}}{j!}+pe^{-\frac{\left(1+\theta \right)\lambda_{1}}{\beta \mu }p}\frac{{\left(\frac{\left(1+\theta \right)\lambda_{1}}{\beta \mu }p\right)}^{j-1}}{(j-1)!}. \end{array}$$

In terms of the definition of PGF, the PGF of $Y_{k}$ is

(A3) $$\begin{array}{cc} G_{Y}\left(z\right) & =E\left(z^{Y_{k}}\right)=\sum_{j=0}^{\infty }p_{j}^{y}z^{j}\\ & =\left(1-p\right)z^{j}e^{-\frac{\left(1+\theta \right)\lambda_{1}}{\beta \mu }p}\frac{{\left(\frac{\left(1+\theta \right)\lambda_{1}}{\beta \mu }p\right)}^{j}}{j!}+pz^{j}e^{-\frac{\left(1+\theta \right)\lambda_{1}}{\beta \mu }p}\frac{{\left(\frac{\left(1+\theta \right)\lambda_{1}}{\beta \mu }p\right)}^{\left(j-1\right)}}{(j-1)!}\\ & =\left(1-p+pz\right)e^{\frac{\left(1+\theta \right)\lambda_{1}}{\beta \mu }p\left(z-1\right)}. \end{array}$$

 □

## Appendix B

**Proof.** When $p\left(1+\frac{\left(1+\theta \right)\lambda_{1}}{\beta \mu }\right)<1$, the queue is stable. According to the queueing theory and properties of stationary distribution, we have πP=π and

$$\pi =\pi_{0}p_{j}^{y}+\sum_{i=1}^{j+1}\pi_{i}p_{j+1-i}^{y},\;j\geq 0.$$

The two sides of the equation are multiplied by $z_{j}$, we can obtain

(A4) $$\begin{array}{cc} G_{L_{k}}\left(z\right) & =\pi_{0}\sum_{j=0}^{\infty }p_{j}^{y}z^{j}+\sum_{j=0}^{\infty }z^{j}\sum_{i=1}^{j+1}\pi_{i}p_{j+1-i}^{y}\\ & =\pi_{0}G_{Y}\left(z\right)+\frac{1}{z}\left[G_{L_{k}}\left(z\right)-\pi_{0}\right]G_{Y}\left(z\right), \end{array}$$

where $G_{Y}\left(z\right)$ is given by Equation (A3). In order to calculate $G_{L_{k}}\left(z\right)$, Equation (A4) can be expressed as

(A5) $$G_{L_{k}}\left(z\right)=\frac{\pi_{0}\left(1-z\right)G_{Y}\left(z\right)}{G_{Y}\left(z\right)-z}=\frac{\pi_{0}\left(1-z\right)}{1-z/G_{Y}\left(z\right)}.$$

According to the properties of the PGF, we have

$$\lim_{z\to 1}G_{L_{k}}^{\prime }\left(z\right)=\frac{\pi_{0}}{1-p-\frac{\left(1+\theta \right)\lambda_{1}}{\beta \mu }p}=1.$$

Therefore, $\pi_{0}=1-p-\frac{\left(1+\theta \right)\lambda_{1}}{\beta \mu }p$. We can calculate $G_{L_{k}}\left(z\right)$ by taking $\pi_{0}$ into Equation (A5). □

## Appendix C

**Proof.** In the first-come first-served queue, the queue length at the packet departure time being transmitted is equal to the number of arriving data packets during the system time of the leaving data packet. Thus,

$$\mathrm{Pr}\{L_{k}=j\}=\sum_{i=j}^{\infty }\mathrm{Pr}\left(T_{k}^{\left(T\right)}=i\right)C_{i}^{j}p^{j}{\left(1-p\right)}^{i-j},$$

where j=0,1,2,⋯,andj≤i. According to the definition of PGF, we have

(A6) $$\begin{array}{cc} G_{L_{k}}\left(s\right) & =E\left(s^{L}\right)=\sum_{j=0}^{\infty }s^{j}\mathrm{Pr}\{L_{k}=j\}\\ & =\sum_{j=0}^{\infty }s^{j}\sum_{i=j}^{\infty }\mathrm{Pr}\left\{T_{k}^{\left(T\right)}=i\right\}C_{i}^{j}p^{j}{\left(1-p\right)}^{i-j}\\ & =\sum_{i=0}^{\infty }\mathrm{Pr}\left\{T_{k}^{\left(T\right)}=i\right\}\sum_{j=0}^{i}C_{i}^{j}{\left(ps\right)}^{j}{\left(1-p\right)}^{i-j}\\ & =\sum_{i=0}^{\infty }\mathrm{Pr}\left\{T_{k}^{\left(T\right)}=i\right\}{\left(1-p+ps\right)}^{i}\\ & =G_{T}\left(1-p+ps\right). \end{array}$$

Combining formulas $G_{L_{k}}\left(z\right)$ in Proposition 5 and $G_{L_{k}}\left(s\right)=G_{T}\left(1-p-ps\right)$ in Equation (A6), and replacing (1−p−ps) with *z*, we can get $G_{T}\left(z\right)$. □

## Appendix D

**Proof.** In order to calculate $E\left(I_{k}T_{k}^{\left(W\right)}\right)$, we define an auxiliary function as

(A7) $$\begin{array}{cc} F(z) & =\sum_{i=1}^{\infty }\mathrm{Pr}\left\{I_{k}=i\right\}z^{i}\sum_{j=0}^{\infty }\mathrm{Pr}\left\{T_{k}^{\left(T\right)}\geq i+j\right\}\\ & =\sum_{j=1}^{\infty }\sum_{i=1}^{j}\mathrm{Pr}\left\{I_{k}=i\right\}z^{i}\mathrm{Pr}\left\{T_{k}^{\left(T\right)}\geq j\right\}\\ & ≐\sum_{j=1}^{\infty }\mathrm{Pr}\left\{T_{k}^{\left(T\right)}\geq j\right\}pz\frac{1-{\left(1-p\right)}^{j}z^{j}}{1-(1-p)z}\\ & ≐\frac{pz}{1-(1-p)z}\left(E\left(T_{k-1}^{\left(T\right)}\right)-\frac{1-G_{T}\left((1-p)z\right)}{1-(1-p)z}\right), \end{array}$$

where the last two {≐} obtained by Lemma 3 [42]. $G_{T}\left(z\right)$ and $E\left(T_{k-1}^{\left(T\right)}\right)$ are given by Equations (25) and (26). Thus, we have

(A8) $$\begin{array}{c} E\left(I_{k}T_{k}^{\left(W\right)}\right)=E\left(I_{k}E\left(\max\left(0,T_{k-1}^{\left(T\right)}-I_{k}\right)\right)\right)\\ =\sum_{i=1}^{\infty }i\mathrm{Pr}\left(I_{k}=i\right)\sum_{j=0}^{\infty }\mathrm{Pr}\left(\max\left(0,T_{k-1}^{\left(T\right)}-i>j\right)\right)\\ =\sum_{i=1}^{\infty }i\mathrm{Pr}\left(I_{k}=i\right)\sum_{j=0}^{\infty }\mathrm{Pr}\left(T_{k-1}^{\left(T\right)}>j+i\right)\\ =\lim_{z\to 1^{-}}{\left(F\left(z\right)\right)}^{\prime }\\ =\lim_{z\to 1^{-}}\frac{p(E\left(T_{k-1}^{\left(T\right)}\right))}{{\left(1-\left(1-p\right)z\right)}^{2}}+\frac{p(1-p)z}{{\left(1-\left(1-p\right)z\right)}^{2}}G_{T}^{\prime }\left(\left(1-p\right)z\right)-\frac{p(1+(1-p)z)}{{\left(1-\left(1-p\right)z\right)}^{3}}\left(1-G_{T}\left(\left(1-p\right)z\right)\right)\\ =\frac{\left(-2+4p+3\frac{\left(1+\theta \right)\lambda_{1}}{\beta \mu }p\right)\frac{\left(1+\theta \right)\lambda_{1}}{\beta \mu }}{2p(1-p-\frac{\left(1+\theta \right)\lambda_{1}}{\beta \mu }p)}+\frac{\left(1-p-\frac{\left(1+\theta \right)\lambda_{1}}{\beta \mu }p\right)\left(e^{\frac{\left(1+\theta \right)\lambda_{1}}{\beta \mu }p}-1\right)}{p^{2}}, \end{array}$$

where $G_{T}\left(\left(1-p\right)z\right)$ and $G_{T}^{\prime }\left(\left(1-p\right)z\right)$ can be calculated by Equation (25). □
